# East/Central/South African Genotype in a Chikungunya Outbreak, Dhaka, Bangladesh, 2017

**DOI:** 10.3201/eid2502.180188

**Published:** 2019-02

**Authors:** Mizanur Rahman, Junya Yamagishi, Rummana Rahim, Abu Hasan, Abu Sobhan

**Affiliations:** Apollo Hospitals Dhaka, Dhaka, Bangladesh (M. Rahman, R. Rahim, A. Hasan, A. Sobhan);; Hokkaido University, Sapporo, Japan (J. Yamagishi)

**Keywords:** chikungunya, dengue, arbovirus, outbreak, Dhaka, Bangladesh, mosquitoborne illness, ECSA, CHIKV, East/Central/South African, febrile illness, viruses

## Abstract

In 2017, an unprecedented increase in febrile illness was observed in Dhaka, Bangladesh. Real-time reverse transcription PCR confirmed that 603 (40.2%) of 1,500 cases were chikungunya fever. Phylogenetic analysis revealed circulation of the non-A226V East/Central/South African genotype of chikungunya virus in Bangladesh.

Chikungunya fever is a mosquito-transmitted viral disease caused by chikungunya virus (CHIKV; genus *Alphavirus*, *Togaviridae*). Since first isolation from a febrile patient in Tanzania in 1952, CHIKV has been responsible for numerous well-documented outbreaks and epidemics in Africa and Southeast Asia, involving hundreds of thousands of cases (*1*). 

CHIKV strains are clustered into 3 separable genotypes: West African, East/Central/South African (ECSA), and Asian. During 1960–1999, outbreaks in Thailand, Cambodia, Vietnam, Myanmar, the Philippines, Malaysia, Indonesia, Pakistan, and India were caused by strains of the Asian genotype (*1*). However, since 2005, massive epidemics in the Indian Ocean islands and the worldwide increase in travel have altered circulating genotypic distribution of CHIKV. Studies have shown that different lineages of CHIKV epidemic strains of the ECSA genotype have expanded locally and spread to new areas in Africa, Europe, Asia, and the Americas, in addition to Africa and Asia (*2*,*3*). Dengue virus (DENV) is another important arbovirus, spread by the bite of the same group of mosquitoes, *Aedes aegypti* and *Ae. Albopictus,* that transmit CHIKV. DENV and CHIKV are often found co-circulating during outbreaks and have an overlapping clinical presentation; thus, misdiagnosis and underreporting of chikungunya infection in dengue-endemic areas is common (*4*).

In Bangladesh, dengue fever was reported in the mid-1960s and dengue hemorrhagic fever in 2000; both diseases are now endemic to Bangladesh (*5*). In contrast, CHIKV infection in Bangladesh was serologically confirmed for the first time in 2008 (*6*). Thereafter, sporadic cases of chikungunya have been reported (*7*); however, in 2017, chikungunya emerged as an important public health issue (*8*). 

Most patients seek healthcare during the acute, febrile phase of the disease, when IgG and IgM titers are typically below the level of detection limits of serologic diagnostic approaches. Therefore, molecular methodologies to detect viral RNA are highly advantageous to detect and differentiate between cocirculating arboviruses and thus facilitate rapid diagnosis and appropriate treatment. 

During June 29–October 31, 2017, a total of 1,500 patients visited Apollo Hospitals Dhaka (Dhaka, Bangladesh) with acute onset of fever (days 1–7 from onset), myalgia, arthralgia, and headache; some patients experienced a maculopapular rash, gastrointestinal symptoms, or both. We collected serum samples from these patients for routine serologic testing and for molecular detection of CHIKV and DENV by real-time reverse transcription PCR (rRT-PCR). We extracted RNA from serum by using QIAamp MinElute Virus Spin Kit (QIAGEN, https://www.qiagen.com) according to the manufacturer’s instructions. We used purified RNA in a 1-step multiplex rRT-PCR test for the simultaneous detection and differentiation of CHIKV and DENV (Fast Track Diagnostics, http://www.fast-trackdiagnostics.com) on the Rotor Gene Q platform (QIAGEN). Among the 1,500 acute-phase serum samples, rRT-PCR confirmed 603 (40.2%) as CHIKV positive, 233 (15.73%) as DENV positive, and 10 (0.66%) as CHIKV and DENV positive (Appendix Table). 

Because of heightened public awareness and government efforts taken to control mosquitoes, the chikungunya cases gradually decreased (Appendix Figure). We then set out to determine the genotype causing the massive CHIKV outbreak. 

By using stored RNA, serum samples, or both from deidentified samples, we performed a 1-step rRT-PCR with the SuperScript III 1-step rRT-PCR system with platinum Taq DNA polymerase (ThermoFisher, https://www.thermofisher.com) and a published oligonucleotide primer set (20F and 20R) targeting the E1 gene (*9*). We randomly selected high-titer viral RNA samples from 32 rRT-PCR–positive samples with a cycle threshold <30. We verified PCR products of correct molecular weight by gel electrophoresis and directly sequenced 4 randomly selected amplicons by using Sanger method. We submitted these sequences to DDBJ (accession nos. LC364266–LC364269). 

In parallel, we added custom index adaptors with a second rRT-PCR test and sequenced them by using MinION sequencer with the R9.5 flowcell (FLO-107) and 1D2 sequencing kit (LSQ-308) (Nanopore Technologies, https://nanoporetech.com), following the manufacturer’s protocol. We determined the consensus sequences of the E1 gene for 27 CHIKV samples with enough reads by using the Canu assembler, even though a couple of inconsistencies were noted between the Sanger and MinION sequences, which probably were attributable to the lower sequencing accuracy of the MinION sequencer. We aligned the sequences obtained with the reference strains available from GenBank and constructed a phylogenetic tree (Figure) by using MEGA7 (https://www.megasoftware.net). 

**Figure:**
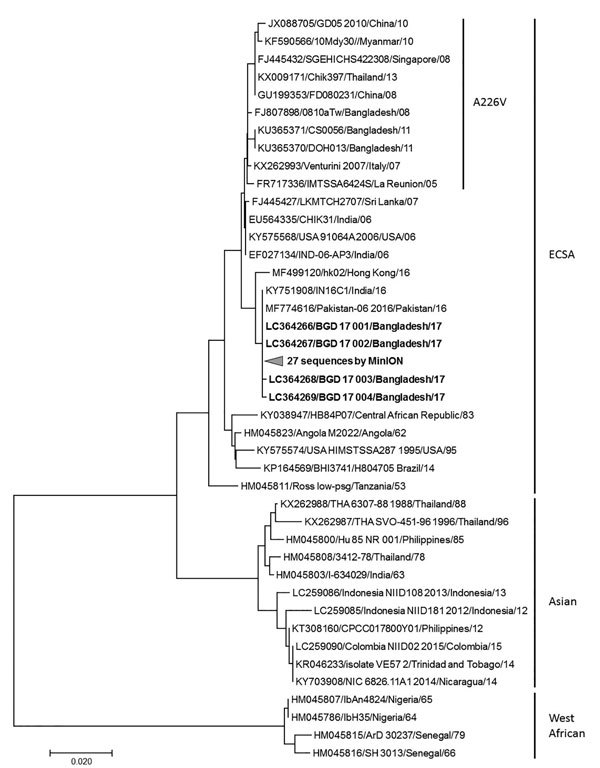
Phylogenetic tree for partial chikungunya virus E1 gene nucleotide sequences with reference strains, Apollo Hospitals Dhaka, Dhaka, Bangladesh, June 29–October 31, 2017. Bold indicates sequences obtained in this study. Representative strains of each genotype are named by accession number, strain name, country of origin, and year of isolation. Scale bar indicates nucleotide substitutions per site.

The results suggested that all the CHIKV E1 sequences obtained from Bangladesh patients in 2017 belonged to the ECSA genotype and did not harbor the A226V mutation, which is different from the previously reported CHIKV strain detected in Bangladesh in 2008 (*10*) and 2011 and more genetically related to the lineage circulating endemically in India and Pakistan in 2016. An extensive genome analysis of recent strains of CHIKV along with research on environmental factors is necessary to better understand the factors underlying the recent outbreak to inform efforts to mitigate potential outbreaks in the future.

Appendix Additional information on East/Central/South African genotype in a chikungunya outbreak, Dhaka, Bangladesh, 2017.
