# Supplementary material for: East/Central/South African Genotype in a Chikungunya Outbreak, Dhaka, Bangladesh, 2017
Source: Emerg Infect Dis. 2019 Feb;25(2):370–2. doi: 10.3201/eid2502.180188 (PMC6346441; doi:10.3201/eid2502.180188)
Supplement: Appendix — Additional information on East/Central/South African genotype in a chikungunya outbreak, Dhaka, Bangladesh, 2017. [file 18-0188-Techapp-s1.pdf]

Article DOI: https://doi.org/10.3201/eid2502.180188

East/Central/South African Genotype in a Chikungunya Outbreak, Dhaka, Bangladesh, 2017

Appendix

Appendix Table. Distribution of RT-PCR confirmed cases of chikungunya and dengue, India*

Age group, y	No. suspected cases	CHIKV+, no. (%)	DENV+, no. (%)	No. coinfections
<1	27	5 (18.5)	5 (18.5)	0
1–10	392	104 (26.5)	56 (14.28)	2
11–20	154	46 (29.8)	33 (21.42)	0
21–30	159	70 (44.2)	31 (19.49)	2
31–40	202	101 (50.0)	38 (18.80)	1
41–50	181	74 (40.8)	40 (22.09)	2
51–60	110	56 (50.9)	14 (12.70)	2
61–70	137	83 (60.5)	10 (7.29)	1
>70	128	62 (48.4)	9 (7.03)	0
Total	1,500	603 (40.2)	233 (15.73)	10

*CHIKV+, chikungunya virus positive; DENV+, dengue virus positive; RT-PCR, reverse transcription polymerase chain reaction.

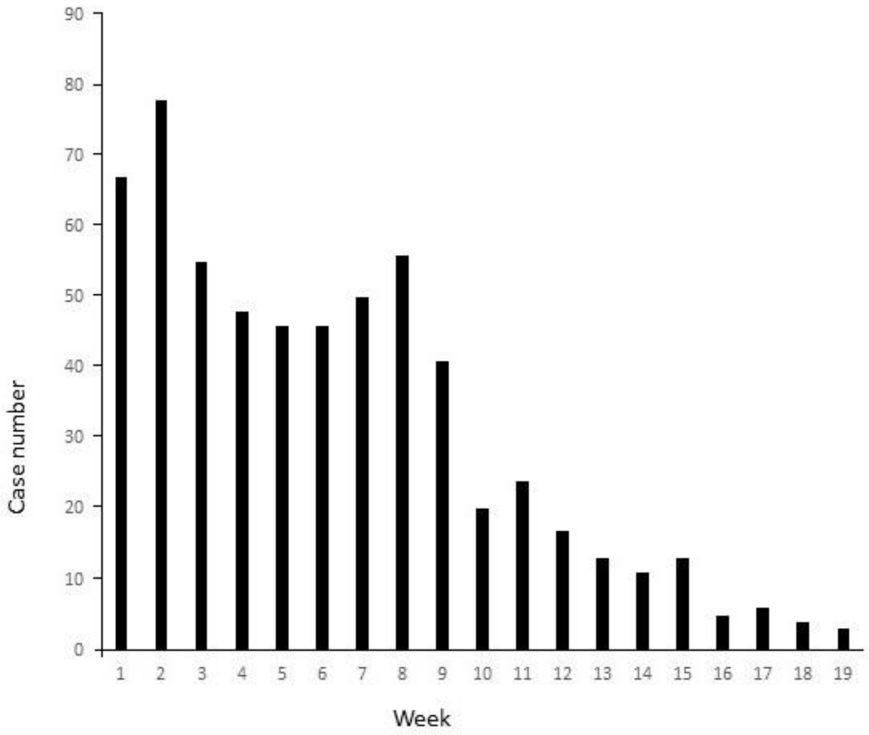

Appendix Figure. Reported cases of chikungunya per week in Apollo Hospitals Dhaka, Bangladesh, June 29–October 31, 2017.
